# Perspectives in Pediatric Ambulatory Anesthesia: Part 3—One Center's Evolving Regional Anesthesia Experience and Outcomes

**DOI:** 10.1002/pan.70255

**Published:** 2026-07-09

**Authors:** David E. Liston, Elizabeth E. Hansen, Jennifer L. Chiem, Sean H. Flack, Daniel K. W. Low, Lynn D. Martin

**Affiliations:** ^1^ Department of Anesthesiology & Pain Medicine Seattle Children's Hospital, University of Washington School of Medicine Seattle Washington USA

**Keywords:** ambulatory surgery, pediatric anesthesia, quality improvement, regional anesthesia, safety

## Abstract

**Introduction:**

Pediatric ambulatory surgery is the dominant model of surgical care in the U.S. and has impacted the practice of regional anesthesia (RA). Pediatric RA's use has expanded as a supplement to general anesthesia with the introduction of ultrasound. We wish to document RA practice changes over the last 14 years in our ambulatory surgery center (ASC).

**Methods:**

A retrospective review of RA practice from 7/2010 to 12/2024 at a free‐standing pediatric ASC was performed. A commercial software system extracted de‐identified, aggregated health data from the system's EMR. Using statistical process control methods, data were processed to distinguish between common and special cause variation.

**Results:**

Incidence of peripheral nerve blocks (PNB) was 13% and increased to 35.4% (associated with ultrasound use and pudendal block standard for penile surgery). The inverse was seen with central neuraxial block (CNB), dropping from 10% of cases to 2%. At baseline 25% of cases received a single injection block, increasing three‐fold (associated with ultrasound/pudendal introductions, COVID‐related reduction in non‐regional cases, and non‐regional case volume recovery). The incidence of continuous catheter cases was 3.7% and decreased twice (associated with COVID and sports medicine catheter program closure). Anesthesia preparation time with RA decreased from 16.8 to 12.5 min (associated with ultrasound introduction, end of COVID restrictions, and sports medicine catheter program closure). The first attempt RA success rate was high (PNB‐90%, CNB‐83%; single injection‐89%, continuous catheter‐96%). PACU maximum pain and opioid use rates decreased for RA and non‐RA patients. Wrong‐site blocks, their root cause analyses, and improvements to the RA timeout are discussed.

**Discussion:**

This review's key findings include an evolution in RA practice, which in conjunction with opioid‐sparing efforts, have driven improvements in clinical outcomes. Our ambulatory pediatric RA practice may not reflect practice seen in others; thus, we do not make inferences outside our facility.

**Abbreviations**
ACS: acute compartment syndrome
ASC: ambulatory surgery center
CNB: central neuraxial block
CPT: current procedural terminology
EMR: electronic medical record
ERP: enhance recovery program
OFA: opioid‐free anesthesia
OR: operating room
PACU: post anesthesia care unit
PDSA: Plan‐Do‐Study‐Act
PNB: peripheral nerve block
PRAN: Pediatric Regional Anesthesia Network
QI: quality improvement
RA: regional anesthesia
RCA: root cause analysis
SCV: special cause variation
SPC: statistical process control
TAP: transversus abdominis plane
US: United States
WSB: wrong‐site block

## 1 Introduction

As highlighted by Vanderhoek et al. [1], pediatric ambulatory surgery is the dominant model of pediatric surgical care in the United States (US). These changes in practice are being driven in part by economic forces, such as insurance mandates and cost savings. There is a high amount of variability seen in these practices and disconnects between research (i.e., publications) and where most pediatric perioperative care is delivered. They also described many challenges and opportunities in the current climate, identifying important and growing knowledge gaps. As discussed in Part 1 (eligibility and outcomes) and 2 (quality outcomes) of these perspective manuscript series, our practice of pediatric ambulatory anesthesia has also evolved significantly [2, 3].

Growth in ambulatory surgery has impacted the field of regional anesthesia (RA); having taken on a larger role in the perioperative, acute pain setting; particularly with the onset of the current opioid crisis [4]. RA provides both surgical anesthesia and postoperative analgesia with minimal systemic side effects; thus improving the quality of postoperative pain control and recovery [5]. Therefore, it is not surprising that RA is taking on an expanded role in ambulatory surgery [6]. New RA agents, adjuncts, and improvements in pharmacokinetic data have been used to reduce pain and support recovery [7, 8]. Detailed assessments of functional outcomes after major orthopedic surgery, rather than pain control alone, are needed to better establish the role of RA [9]. No difference in complications or clinical outcomes with or without RA in adults undergoing ambulatory shoulder surgery has been reported [10].

The history and benefits of RA in children are well described [11, 12, 13]. The use of RA in children has expanded dramatically with the shift to its use as a supplement to general anesthesia and subsequently the introduction of ultrasound to allow direct visualization of relevant anatomy [14]. Barnett's review discusses many of the controversies in pediatric RA: awake versus asleep administration, compartment syndrome, test dosing, air versus saline for loss of resistance during epidural placement, etc. Cumulative experience and evidence have led to the publication of widely recognized guidelines of care for pediatric RA [15].

Based on concerns regarding the safety of RA in children, investigators have published the incidence of complication rates in children from both the US and Europe. The first report from the Pediatric Regional Anesthesia Network (PRAN) found no mortality or long‐term (> 3 month) complications after nearly 15 000 regional blocks [16]. Continuous catheter blocks had a higher complication rate than single injection blocks. A follow up study of over 100 000 blocks showed no permanent neurologic complications and a low risk for local anesthetic systemic toxicity (most occurring in infants), infections, and hematomas [17]. The most common complication was benign catheter‐related failures (4%). Findings from Europe reveal a lower frequency of RA and a higher reliance on central as opposed to peripheral nerve blocks (PNB) [18]. Large variability in practices was seen.

### 1.1 Specific Aim

As described in Parts 1 and 2, we chose to complete a comprehensive retrospective review of our process, outcome, and balancing metrics contained in our electronic medical record (EMR) from our pediatric ambulatory surgery center (ASC) since its opening on 7/1/2010 through 12/31/2024. In Part 3 we detail the significant evolution in our single‐center ambulatory pediatric RA practices and outcomes.

## 2 Methods

A comprehensive review of our methods was described in Part 1 [2], we summarize here.

### 2.1 Context

Seattle Children's is a pediatric health system affiliated with the University of Washington with 345 licensed beds, 24 operating rooms (ORs), and 4 procedure suites. Our institution utilizes Lean principles (standardized practice, waste minimization, visual tracking of information, and continuous iterative improvement using Plan‐Do‐Study‐Act [PDSA] cycles) to enhance healthcare outcomes [2, 3]. Leaders and staff update and use standard anesthesia protocols based on evidence and consensus‐driven methods. Protocol compliance is facilitated by case‐specific EMR templates. The ability to create custom patient cohorts and visualize key process and outcome metrics from the EMR promotes rapid understanding and increased rates of PDSA cycle improvements [19, 20].

The free‐standing pediatric ASC affiliated with Seattle Children's was opened in 7/2010 with the intention to serve as a site where clinicians and nursing staff develop standardized protocols derived from literature and consensus to enhance clinical outcomes. Multimodal analgesia has been a foundation of this practice, where regional anesthesia supplements the general anesthetic whenever possible. The healthy patient population, low‐acuity cases with high volumes, standardized clinical practices, accessible process and outcomes data, and the use of Lean methods all support the goal of continuous process and outcome improvements.

### 2.2 Measures

AdaptX (Seattle, WA, USA) is a software system that extracts de‐identified, aggregated, and continuously (daily) updated health data from the system's EMR. The data were processed and presented in statistical process control (SPC) charts allowing clinicians to distinguish between common cause/random variation (noise) and non‐random or special cause variation (SCV) [19]. Shewhart's theory of variation was used to identify SCV. By SPC convention, control limits are set at 3 sigma above and below the weighted mean (centerline). A run of 12 points without SCV is representative of a stable process [20].

SPC chart types have been previously described [2, 19]. The P‐chart was used for the incident of RA, non‐RA, PNB, central neuraxial blocks (CNB), single injection blocks, continuous catheter blocks, and ultrasound guidance EMR coding. A C‐chart was used for quarterly surgical case counts and quarterly PNB case counts. An X‐bar chart was used for average anesthesia preparation time (defined as time from anesthesia start to anesthesia ready), post anesthesia care unit (PACU) maximum pain score, and opioid administration rate. Each X‐bar chart has a paired S‐chart that displays the standard deviation (variability) of this data. Prime corrections (used for data with over‐dispersion) [19] were utilized for quarterly surgical case counts, PNB case counts, incidence of PNB, CNB, single injection blocks, catheter blocks, and ultrasound guidance coding. Any change noted in our results section is associated with SCV.

### 2.3 Ethical Consideration

The series of quality improvement (QI) projects and this specific summary were submitted to Seattle Children's Institutional Review Board. The board considered this QI work. No further review was required.

## 3 Results

### 3.1 ASC Regional Focus and Timeline

Since its opening, the ASC has been our site for innovation via continuous improvement for the organization, including the practice of pediatric RA. The list of RA events and milestones is found in Table 1 and their associated timeline in Figure 1. Extensive use of RA was introduced with the opening as part of the multimodal analgesia and opioid‐sparing anesthesia protocols.

**TABLE 1:** ASC and regional anesthesia events.

Event	Description	Date
ASC Open	OR1 or OR2 running weekdays	7/20/2010
Never Event #1	Wrong‐Site Block	2/2014
Air Flow Renovations	All ORs closed for airflow renovations and re‐balancing	5/3/2015
Ultrasound Intro	Ultrasound machine purchased for PNB placement	8/1/2016
Pudendal Trial	Ultrasound guided Pudendal block trial	1/1/2019
Center‐wide OFA Protocol	OFA protocol launched for all procedures	1/4/2019
Never Event #2	Wrong‐Site Block	3/2019
Pudendal Policy	Pudendal block standard policy launched	9/1/2019
COVID OR Closure	State Department of Health (DOH) mandates ASC closures for COVID pandemic	3/13/2020
ERP Phase 1	Implement: pre‐op analgesia/carbo liquid; standard rescue post‐op antiemetic Enhance: pre‐op screen/education; opioid‐sparing analgesia, early feeding	1/25/2021
ERP Phase 2	Implement: normothermia, euvolemia processes Enhance: opioid‐sparing analgesia	4/2/2021
ERP Phase 3	Implement: pre‐op strengthening (‘pre‐habilitation’) process Enhance: pre‐op screening, prophylactic antibiotics, opioid‐sparing analgesia	7/19/2021
ERP Phase 4	Dexmedetomidine in PACU, sustainment of data monitoring	7/18/2022
PNB Catheter Closed	PNB catheter placement stopped for Sport Medicine Program	7/1/2023

Abbreviations: ASC, ambulatory surgery center; EMR, electronic medical record; ERP, enhanced recovery program; OFA, opioid‐free anesthesia; PNB, peripheral nerve block.

**FIGURE 1:**
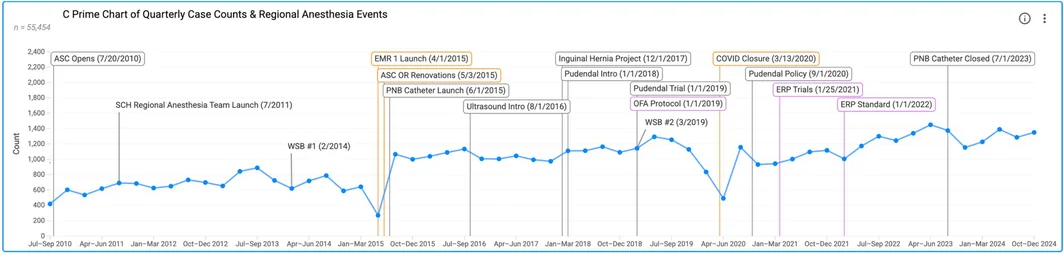
C prime chart of quarterly case counts & regional anesthesia events. Note the details for the annotations for select regional anesthesia events and milestones are defined in Table 1.

### 3.2 Methodologic Evolution

The practice of pediatric RA at our ASC has changed significantly over the last 14 years. PRAN previously reported a large variation in local anesthetic dose within and between hospitals for both caudal CNB and PNB [21, 22]. The percentage of patients receiving RA is displayed in Figure 2. Slightly over 20% of patients received RA at baseline, increasing in 1/2017 after the introduction of ultrasound technology and again in 1/2020 with COVID‐19 and the associated decrease in non‐regional (otolaryngology) cases. Our PNB quarterly case counts and incidence of PNB and CNB are seen in Figure S1 and Figure 3A,B, respectively. The incidence of PNB was 13% at baseline and increased to 21.2% in 1/2018 (associated with ultrasound introduction) and to 35.4% in 10/2020 (associated with the launch of pudendal blocks as the standard for penile surgery). The inverse was seen with CNB, dropping from a baseline of 10% to 2%.

**FIGURE 2:**
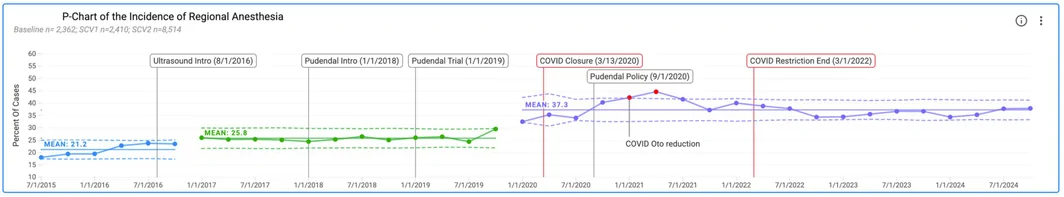
P‐chart of the incidence of regional anesthesia. Cohorts are separated only when SCV is seen. Annotations indicate internal and external events associated with changes in incidence of regional anesthesia. Red dots represent single point SCV likely associated with the reduction in otolaryngology (non‐RA) case volumes.

**FIGURE 3:**
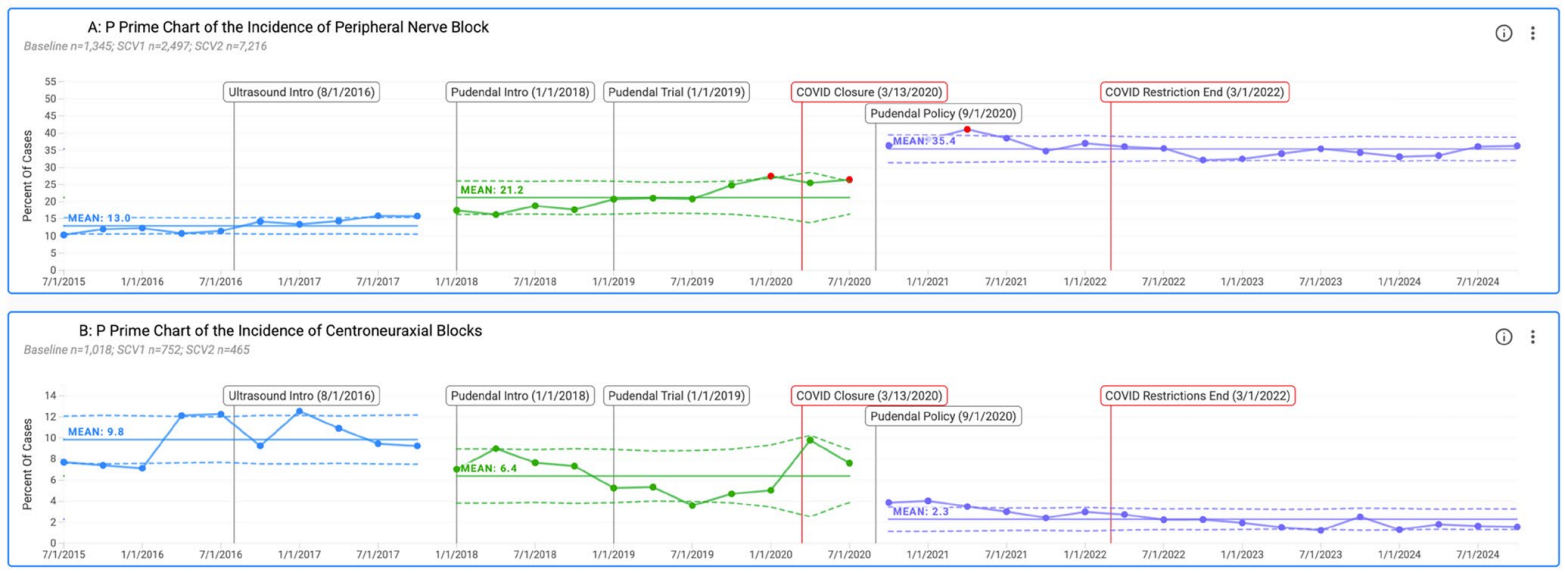
(A) P prime chart of the incidence of peripheral nerve block and (B) P prime chart of the incidence of centroneuraxial blocks. Cohorts are separated only when SCV is seen. Annotations indicate internal and external events associated with changes in incidence of peripheral and central neuraxial nerve blocks. Red dots represent single point SCV.

#### 3.2.1 Block Technique: Single Shot vs. Catheter

Case demographic data for block type and technique are listed in Table 2. Block techniques (single injection versus continuous catheter) also changed over time (Figure 4A,B). 22% of cases received a single injection RA at baseline, increasing to 24.9% in 1/2018 (associated with ultrasound technology introduction), 36.8% in 1/2020 (associated with COVID‐19 reduction in non‐RA cases and launch of the pudendal policy), and decreased slightly to 35.6% in 1/2023 (associated with return of non‐RA case volumes). The incidence of continuous catheter cases was low at baseline (3.7%) and decreased to 1.5% in 10/2020 (associated with COVID) and to 0.2% in 7/2023 when the sports medicine catheter program was closed.

**TABLE 2:** Regional anesthesia case demographics and details.

Characteristic	PNB	CNB	Single injection	Catheter
	(*n* = 11 058)	(*n* = 2235)	(*n* = 12 880)	(*n* = 1017)
Age group				
< 1 year	1739 (15.7%)	1138 (50.9%)	2867 (22.3%)	6 (0.6%)
1–3 years	2113 (19.1%)	1087 (48.6%)	3190 (24.8%)	6 (0.6%)
4–5 years	1275 (11.5%)	8 (0.4%)	1277 (9.9%)	9 (0.9%)
6–12 years	2962 (26.8%)	2 (0.1%)	2874 (22.3%)	193 (19%)
13–18 years	2817 (25.5%)		2540 (19.7%)	765 (75.2%)
> 18 years	152 (1.4%)		132 (1%)	38 (3.7%)
Weight group				
3–5 kgs	4 (0.1%)	31 (1.4%)	35 (0.3%)	
6–10 kgs	2161 (19.5%)	1388 (62.1%)	3535 (27.5%)	10 (1%)
11–20 kgs	2869 (26%)	811 (36.3%)	3671 (28.5%)	7 (0.7%)
21–30 kgs	1445 (13.1%)	4 (0.2%)	1428 (11.1%)	42 (4.1%)
31–40 kgs	782 (7.1%)	1 (0.04%)	762 (5.9%)	56 (5.5%)
41–50 kgs	764 (6.9%)		720 (5.6%)	113 (11.1%)
51–60 kgs	1007 (9.1%)		904 (7%)	252 (24.8%)
61–70 kgs	821 (7.4%)		755 (5.9%)	214 (21%)
71–80 kgs	571 (5.2%)		515 (4%)	154 (15.1%)
81–90 kgs	288 (2.6%)		250 (1.9%)	85 (8.4%)
91–100 kgs	185 (1.7%)		161 (1.2)%	44 (4.3%)
> 100 kgs	161 (1.3%)		144 (1.1%)	40 (4%)
Gender				
Male	8171 (73.9%)	2131 (95.4%)	10 110 (78.5%)	487 (47.9%)
Female	2879 (26.1%)	102 (4.6%)	2768 (21.5%)	530 (52.1%)
ASA physical status				
1	7028 (63.9%)	1512 (67.7%)	8363 (64.9%)	602 (59.2%)
2	3853 (35%)	709 (31.7%)	4381 (34%)	404 (39.7%)
3	122 (1.1%)	11 (0.5%)	128 (1%)	11 (1.1%)
Unknown	3 (0.03%)	3 (0.1%)	7 (0.05%)	
Surgical specialty				
Urology	5291 (47.7%)	1846 (82.2%)	7116 (55.1%)	14 (1.4%)
Orthopedics	3632 (32.8%)	33 (1.5%)	3277 (25.3%)	999 (98%)
Surgery	1951 (17.6%)	364 (16.3%)	2310 (17.9%)	5 (0.5%)
Plastics	215 (1.9%)	1 (0.04%)	215 (1.7%)	1 (0.1%)
Block name				
Pudendal	3942 (35%)		3950 (32.4%)	
Ilioinguinal‐iliohypogastric	2101 (18.7%)		2101 (17.2%)	
Sciatic	2310 (20.5%)		1670 (13.7%)	640 (50.3%)
Adductor canal	1363 (12.1%)		1207 (9.9%)	588 (46.2%)
Rectus Sheath	977 (8.7%)		977 (8%)	
Supraclavicular	566 (5%)		529 (4.3%)	39 (3.1%)
Epidural				
Caudal		2230 (99.8%)	1763 (14.5%)	1 (0.08%)
Lumbar/Thoracic		4 (0.2%)		4 (0.3%)
Spinal		1 (0.04%)	1 (0.008%)	
Number of attempts				
1	10 019 (90.3%)	1849 (82.7%)	11 488 (89.2%)	979 (90.5%)
2	905 (7.8%)	266 (11.9%)	1137 (8.8%)	88 (8.1%)
3 or more	225 (1.9%)	123 (5.4%)	346 (2%)	15 (1.4%)

Abbreviations: CNB, central neuraxial block; PNB, peripheral nerve block.

**FIGURE 4:**
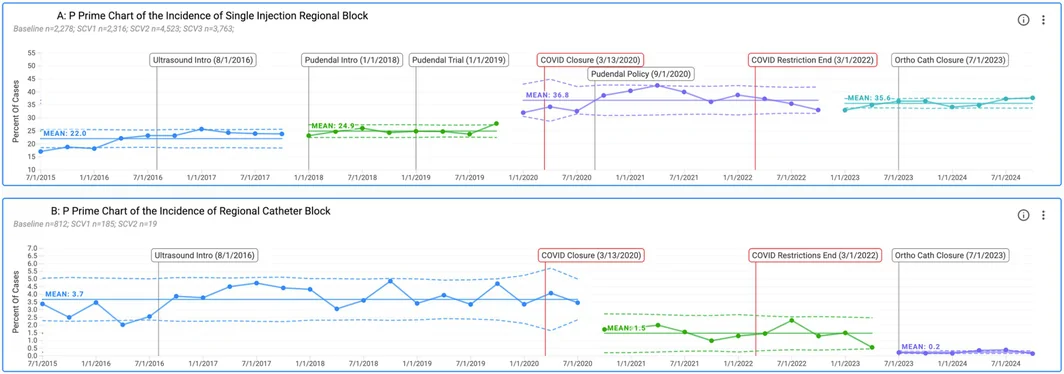
(A) P prime chart of the incidence of single injection regional block and (B) P prime chart of the incidence of regional catheter block. Cohorts are separated only when SCV is seen. Annotations indicate internal and external events associated with changes in the incidence of single injection and continuous catheter nerve blocks.

Johnstone et al. have recently explored strategies to optimize PNB for both single injection and continuous catheters [23]. We had previously reported no difference between femoral versus adductor canal continuous catheters for knee arthroscopy and repairs in adolescents [24]. Walker et al. have reported on the safety of PNB catheters from PRAN [25]. Catheter malfunction was the most common complication and there were no reported persistent neurologic problems, infections, or local anesthetic toxicity. Longer duration of catheter use was associated with the risk of infection. Similarly, Chun et al. reported that pediatric RA is safe and not associated with neurologic complications [26].

The introduction of ultrasound technology revolutionized the practice of RA and enhanced safety in both adults and children [27, 28]. Bosenberg described the use of ultrasound to conduct advanced truncal nerve blocks (lumbar plexus and erector spinae) in children [29]. With the introduction of ultrasound technology at our ASC in 8/2016, anesthesiologists began documenting the use of ultrasound via billing Current Procedural Terminology (CPT) code 76 942 (Figure S2); nearly 80% of blocks are associated with this code. There was a reduction associated with the transition to a second commercial EMR vendor as well as the COVID‐19 pandemic. We suspect there is under documentation of this service as our practice guidelines have not changed.

As noted previously, there is typically large variation in RA local anesthetic dosing [21, 22]. As an institution that develops and follows standard care protocols, we have limited our local anesthetic to only ropivacaine (0.2% or 0.5%). Approximately 98% of our blocks are completed with ropivacaine only (adjuncts are not typically added). This represents 10 883 PNBs, 2188 CNB, 12 615 single injections, and 998 continuous catheters. The top 6 block sites in decreasing order were pudendal (3852), caudal (2182), ilioinguinal‐iliohypogastric (2051), sciatic (1299), adductor canal (1220), and supraclavicular (542).

### 3.3 Controversies

#### 3.3.1 Asleep

After a published report by Bromage and Benumof [30], the practice of placing regional anesthesia in anesthetized children to improve compliance and safety has been widely debated. Large‐scale studies in both the US and French‐language centers have demonstrated this to be a safe and reliable practice [31, 32, 33].

#### 3.3.2 Acute Compartment Syndrome

Another concern regarding pediatric RA is masking the signs and symptoms of postoperative acute compartment syndrome (ACS) in children. Two extensive reviews of this topic suggest that low concentration local anesthetics do not typically mask ACS [34, 35]. The seven pillars for early detection of ACS include recognition and vigilance for the following:

#### 3.3.3 Block Placement

○ Perform a test dose.
○ Utilize minimum concentration/dose of local anesthetic for PNBs at high risk for ACS.

#### 3.3.4 Post‐Block

○ Closer monitoring for patients with additional risk factors (e.g., coagulation disorder).
○ Monitor for pain and paresthesia/paresis.
○ Closer monitoring for patients with pathologies (e.g., trauma, bleeding) in the compartment.
○ Disproportionate pain requires immediate diagnostic steps.
○ Diagnosis requires clinical examination and when necessary, intracompartmental pressure monitoring.

Early detection of inadvertent intravascular injection and local anesthetic systemic toxicity are difficult to detect in the anesthetized child. Both blood in the needle and injection of small doses of epinephrine have been described, but are associated with low sensitivity [36]. This unreliability and delay in detection suggest that safety can be further enhanced with incremental, fractioned dosing with intermittent aspiration [37]. Based upon available evidence, the European and American Societies of Regional Anesthesia published joint practice advisory guidelines suggesting that placement of RA under general anesthesia in children is safe and the standard of care. Use of test dosing is discretionary, and there is no evidence that RA increases risk for ACS or delays its diagnosis [38].

### 3.4 Outcomes

It is paramount to objectively assess clinical outcomes of RA in all domains of quality. We will focus on the three most important.

#### 3.4.1 Efficiency

Due to time spent placing RA blocks, surgeons may request placement after surgery to provide postoperative pain relief without delays in surgical processes. This practice obviously precludes the benefits of RA and reduced general anesthetic requirements when it is used to supplement general anesthesia. Thus, this is not our standard of care. To address surgical concerns with preoperative time spent performing RA, we monitor anesthesia preparation time (defined as time from anesthesia start to anesthesia ready for handoff to the surgical team). Anesthesia preparation time and standard deviation are shown in Figure S3A,B. Average preparation time decreased to 15.9 min in 1/2017 after introduction of ultrasound technology, 13.9 min in 7/2022 after removal of COVID‐19 restrictions, and to 12.5 min in 7/2024 1 year after ending the sports medicine catheter program. The time savings of > 4 min (25.6%) is considered clinically significant by our surgeons. There were also several decreases in variability seen with ultrasound introduction, COVID‐19 restrictions and their ending, and again with the continuous catheter program closure.

A second process measure we monitor to assure both efficiency and safety is the number of block placement attempts as recorded in the EMR by the anesthetist (Table 2). First attempt success rate was 90% for PNB, 83% for CNB, 89% for single injection, and 96% for continuous catheter. This rate was driven by the high frequency of block placement for all ASC anesthesiologists and limited teaching requirements (no resident trainees and fellow trainees are present only 24 weeks/year).

#### 3.4.2 Effectiveness

Postoperative pain has historically been seen as the primary effectiveness measure for RA. Xie et al. completed a retrospective cohort assessment of RA in patients under 18 years of age [39]. They found use of RA (both single injection and continuous catheter blocks) was associated with opioid‐free anesthesia and opioid‐free PACU stays. Catheter cases were associated with reduced PACU and inpatient opioid use in the first 24 h postoperatively, but no difference was seen after this period. Single injection blocks were not associated with improved pain scores or reduced opioid use.

We also routinely monitor these same metrics as effectiveness measures. The average PACU maximum pain score showed SCV for RA cases in Figure S4A,B and non‐regional cases in Figure S4C,D. The average maximum pain score (0–10) is 2.8 at baseline and decreased to 2.2 in 4/2021 during the enhanced recovery program trials and to 1.9 in 7/2023 associated with the sports medicine continuous catheter program closure. Only one decrease in pain score variability was seen in 1/2022. Non‐RA patients also had decreased maximum pain scores to 2.1 in 7/2020 after opioid‐free anesthesia policy launch, while variability had a decrease in 7/2021 associated with enhanced recovery trials. The average PACU opioid administration rate for RA patients is shown in Figure S5A and non‐RA patients in Figure S5B. The incidence of PACU opioid administration decreased significantly from baseline (12.1%) to 9% in 1/2019 associated with launching an opioid‐free anesthesia (OFA) policy, to 5.1% in 7/2021 associated with the enhance recovery program (ERP) trials, and to 4.7% in 7/2023 associated with the orthopedic catheter program closure. Patients not receiving RA also had decreased PACU opioid administration associated with OFA policy launch (1/2019) and after ERP trials (10/2021). The improvements in PACU pain scores and opioid use rates in both RA and non‐RA patients were facilitated by institutional commitments to multimodal analgesia and opioid sparing techniques as part of enhanced recovery efforts [3].

#### 3.4.3 Safety

While we have already discussed RA safety risks in children, several deserve more discussion. Placement of RA blocks in patients receiving antithrombotic or thrombolytic therapy has been extensively studied. The American Society of Regional Anesthesia and Pain Medicine has published evidence‐based guidelines on this topic, the fifth edition in 2025 [40]. These guidelines include a procedural anticoagulation management checklist to assist clinicians' assessment of patients with higher risk for bleeding complications. However, they don't address the nuances of anticoagulation management in children, such as age‐dependent heparin half‐life and use of laboratory monitoring, including heparin activity, drug, and anti‐Xa levels. To improve safety, a hospital guideline for children needing regional anesthesia and antithrombotic agents was developed in 2022.

Although infectious complication rates in children receiving RA are low, use of sterile barrier techniques to reduce risk for infectious complications is recommended [16, 17]. Mandler et al. completed a survey of PRAN principal investigators assessing use of sterile barrier techniques [41]. Several areas of practice variability were noted (e.g., utilization of aseptic ultrasound probe cover, sterile gown/towels/drapes). Masaracchia et al. published an educational review focused on potential error traps in RA [42]. They discussed five commonly avoidable error traps while providing RA for children: (1) failure to confirm intended block site, (2) failure to recognize ultrasound artifacts and optimize images, (3) failure to recognize when RA is an acceptable option, (4) failure to implement an alternative imaging technique in patients with challenging anatomy, and (5) failure to recognize disease states with anatomy that may require alternative blocks. The first error trap can lead to a wrong‐site block (WSB); a potentially serious reportable “never” event.

### 3.5 Never Events—Wrong‐Site Blocks

While much of the attention surrounding wrong‐site procedures has focused on wrong‐site surgery, wrong‐site nerve blocks are more common [43, 44]. Wrong‐site blocks place patients at unnecessary risk for block‐related complications and could potentially increase hospital costs and length of stay. The incidence of WSB has been reported to be 1.28 (95% CI 0.43–2.13) per 10 000 patients, more commonly occurring with femoral blocks, and typically associated with policy violations [43]. A systematic literature review found a similar incidence (0.52–5.07 per 10 000 blocks) [44]. The most mentioned contributing factors were time pressure, personnel factors, and lack of site marking. Changing care processes, including block checklists, timeouts, and block site marking, have been recommended to lessen the risk of wrong‐site blocks [45, 46]. The Society for Pediatric Anesthesia quality and safety committee developed a pediatric RA timeout checklist containing 14 safety elements. A simulation study has shown that training and use of the checklist increased both the number of safety items completed and how frequently the team performed or aborted the block as expected [47].

With this background, we will review two WSB events that occurred at our ASC. This represents 2 WSB in 13 895 block placements (an incidence of 1.44 per 10 000 blocks), in line with other published reports [43, 44].

#### 3.5.1 WSB Event 1 (2014)

A 4‐year‐old was scheduled for umbilical hernia and LEFT hydrocele repairs as documented in the clinic notes and confirmed on the surgical consent. The surgeon marked the RIGHT side (verbally confirmed by the parents and witnessed by the anesthesiologist). This discrepancy was not detected with either the surgical sign‐in or regional timeout checklists. The anesthesiologist placed a RIGHT sided transversus abdominis plane (TAP) block. At the time of operative site exposure and surgical timeout, the error was identified. The parents were immediately informed, and a LEFT sided TAP block was placed by the anesthesiologist prior to the start of surgery. The surgery was completed, and the patient was discharged home.

A formal Root Cause Analysis (RCA) was performed. Although correct procedures were followed, the root cause was the failure to confirm laterality on BOTH the surgical consent and surgical patient marking. The RA timeout checklist was modified such that the nurse issues all challenges and the team responds, thereby forcing the anesthesiologist to verify block site laterality with both the surgical consent and surgical site marking (Table S1). The revised checklist was presented to the team, announced at morning huddles, and introduced into practice.

#### 3.5.2 WSB Event 2 (2019)

An 11‐year‐old with developmental delay and behavioral issues was scheduled for RIGHT knee arthroscopy with tibial eminence fixation. The surgical site was confirmed on the consent, by the parents, and the patient was appropriately marked. The patient displayed anxiety and expressed “feeling cold”. The safety sign‐in checklist with surgical site confirmation was completed and anesthesia induction commenced. The regional timeout was completed followed by a brief delay for fellow education. During this delay the patient and surgical site mark were covered with a warm blanket. Upon proceeding, the attending anesthesiologist uncovered the LEFT leg, prepped the skin, and placed the block. The nurse, whose back was turned to complete documentation, turned shortly after block placement and recognized the wrong limb was blocked. The anesthesiologist immediately disclosed the error to the parents and then placed the block on the correct (RIGHT) limb. The surgery was completed, and the patient was discharged home.

Another formal RCA was completed with the root cause being that no block site marking was required. Additional proximate causes included production pressure, distractions, fatigue, and a time lag between completion of the safety checklist and placement of the regional block. Actions taken included modification of our regional timeout checklist to include block site marking (Table S2), revised room set‐up to always facilitate staff line of sight and altered workflow processes with timeout done immediately prior to block placement. Parallel work by team members was discouraged during these critical events (e.g., block placement). A ropivacaine maximum dose job aid was introduced to lessen mental workload while increasing dosing accuracy and team awareness (Table S3). These modifications were presented to the team, announced at morning huddles, and introduce into practice. We have subsequently completed 9492 RA blocks without WSB recurrence.

## 4 Discussion

### 4.1 Summary

A summary of our key findings from our RA review includes: (1) there has been an evolution in RA practice at our pediatric ASC, (2) these changes have supported our opioid‐sparing efforts, and (3) in part have driven improvements in clinical outcomes. Our ambulatory pediatric RA practice may not reflect actual practice seen in the community centers where most pediatric perioperative care is provided.

### 4.2 Strengths

As previously stated [2, 3, 48], the use of easily accessible, continuously updated real‐world data in a real‐time manner allowed the teams to rapidly complete multiple PDSA improvement cycles. These improvement efforts occur in a stable and sustained learning culture [49].

### 4.3 Weaknesses

These QI initiatives have several limitations as described in part 1 [2]. SPC is our primary QI analytical tool that provides a visual display of changes over time, allowing prompt local decision making [19, 20]. We did not perform any supplementary statistical analyses [50], and are not making inferences about populations outside our facility. Pitfalls of prospective, observational QI study designs have been described [51].

This report is restricted to a single pediatric center's experience and uses QI methods and statistics, thus not generalizable. Our staff are pediatric‐trained and experienced. The surgical case mix is determined by the local surgeons and ASC‐approved procedures. As previously described [2], there are center‐specific facility features that influenced our model of care and unique learning cultural characteristics that are uncommon, difficult to create, and sustain [48, 49, 52]. There remains a high amount of variability in published pediatric RA practices [16, 17, 18, 21, 22] and disconnects between published research from academic centers and the clinical sites where most pediatric perioperative care is delivered (e.g., community ASCs) [53].

## 5 Conclusions

The key findings from this single‐center RA review include an evolution in RA practice over the last 14 years, which in conjunction with opioid‐sparing efforts, have driven improvements in clinical outcomes (pain and opioid use). Our ambulatory pediatric RA practice may not reflect actual practice seen in community centers where most pediatric perioperative care is provided; thus we do not make inferences about populations outside our facility. The ease of data access, its transparent use, and the staff mindset embracing continuous change have supported the creation of a learning healthcare system at our ASC.

## Author Contributions

David E. Liston and Jennifer L. Chiem: These authors helped with the acquisition and interpretation of data, participated in the drafting and revision of the content, approved the final version, and agreed to be accountable for all aspects of the work. Elizabeth E. Hansen, Sean H. Flack, Daniel K.W. Low and Lynn D. Martin: These authors helped with the project design, acquisition and interpretation of data, participated in the drafting and revision of the content, approved the final version, and agreed to be accountable for all aspects of the work.

## Funding

The authors have nothing to report.

## Ethics Statement

The Institutional Review Board from Seattle Children's reviewed and waived the need for informed consent.

## Conflicts of Interest

AdaptX is the institution's preferred software tool for EMR QI data extraction and analyses. Daniel Low is founder/CEO and Lynn Martin is CMO of AdaptX. They and David Liston are equity owners of AdaptX. No other authors have any conflicts to report.

## Supporting information


**Figure S1:** C prime chart of the peripheral nerve block quarterly case counts. Cohorts are separated only when SCV is seen. Annotations indicate internal and external events associated with changes in peripheral nerve block case counts. Red dot represents single point SCV.
**Figure S2:** P prime chart of the incidence of ultrasound guidance PNB coding. Cases identified via use of Current Procedural Terminology code for ultrasounds guidance for nerve block (CPT code 76 942). Cohorts are separated only when SCV is seen. Annotations indicate events associated with changes of incidence in ultrasound guided coding. Red dots represent single point SCVs. The blank (ghosted) points represent time when no ultrasound machine was available.
**Figure S3:** (A) X‐bar chart of the average anesthesia prep time with regional block. Anesthesia preparation time is defined as time from anesthesia start to anesthesia ready time. Cohorts are separated only when SCV is seen. Annotations indicate internal and external events associated with changes in peripheral nerve block case counts. Red dots represent single point SCVs. (B) S‐Chart of the Standard Deviation of Anesthesia PrepTime. Anesthesia preparation time is defined as time from anesthesia start to anesthesia ready. Cohorts are separated only when SCV is seen. Annotations indicate internal and external events associated with changes in peripheral nerve block case counts. Red dots represent single point SCVs.
**Figure S4:** (A) X‐bar chart of the average PACU max pain score—regional anesthesia. Cohorts are separated only when SCV is seen. Annotations indicate internal and external events associated with changes in peripheral nerve block case counts. Red dots represent single point SCV. (B) S‐Chart of the Standard Deviation of PACU Max Pain Score—Regional Anesthesia. Cohorts are separated only when SCV is seen. Annotations indicate internal and external events associated with changes in peripheral nerve block case counts. Red dots represent single point SCV. (C) X‐bar Chart of the Average PACU Max Pain Score—No Regional. Cohorts are separated only when SCV is seen. Annotations indicate internal and external events associated with changes in peripheral nerve block case counts. Red dots represent single point SCV. (D) S‐Chart of the Standard Deviation of PACU Max Pain Score—No Regional. Cohorts are separated only when SCV is seen. Annotations indicate internal and external events associated with changes in peripheral nerve block case counts. Red dots represent single point SCV.
**Figure S5:** (A) P‐Chart of the IV Opioids Given in PACU Rate—Regional Anesthesia. Cohorts are separated only when SCV is seen. Annotations indicate events associated with changes in peripheral nerve block case counts. Red dots represent a single point SCV. (B) P‐Chart of the IV Opioids Given in PACU Rate—No Regional. Cohorts are separated only when SCV is seen. Annotations indicate events associated with changes in peripheral nerve block case counts. Red dots represent a single point SCV.

**Table S1:** Regional anesthesia timeout checklist (version 2).

**Table S2:** Regional anesthesia timeout checklist (current version).

**Table S3:** Ropivacaine maximum dose.

## Data Availability

The datasets used and/or analyzed for these quality improvement initiations are available from the corresponding author upon reasonable written request.
