# Supplementary material for: Perspectives in Pediatric Ambulatory Anesthesia: Part 3—One Center's Evolving Regional Anesthesia Experience and Outcomes
Source: Paediatr Anaesth. 2026 Jul 9;36(11):1341–51. doi: 10.1002/pan.70255 (PMC13639202; doi:10.1002/pan.70255)

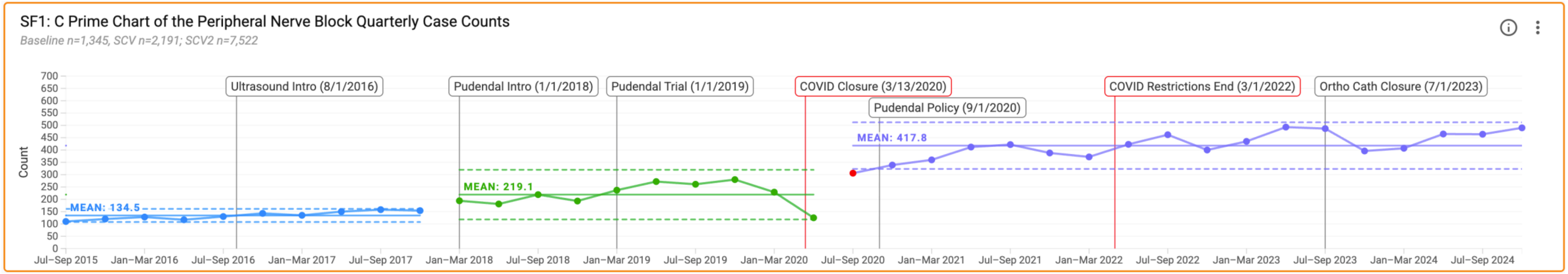
SF1: C Prime Chart of the Peripheral Nerve Block Quarterly Case Counts
Baseline n=1,345, SCV n=2,191; SCV2 n=7,522
Count
700
650
600
550
500
450
400
350
300
250
200
150
100
50
0
Ultrasound Intro (8/1/2016)
Pudendal Intro (1/1/2018)
Pudendal Trial (1/1/2019)
COVID Closure (3/13/2020)
Pudendal Policy (9/1/2020)
COVID Restrictions End (3/1/2022)
Ortho Cath Closure (7/1/2023)
MEAN: 134.5
MEAN: 219.1
MEAN: 417.8
Jul–Sep 2015
Jan–Mar 2016
Jul–Sep 2016
Jan–Mar 2017
Jul–Sep 2017
Jan–Mar 2018
Jul–Sep 2018
Jan–Mar 2019
Jul–Sep 2019
Jan–Mar 2020
Jul–Sep 2020
Jan–Mar 2021
Jul–Sep 2021
Jan–Mar 2022
Jul–Sep 2022
Jan–Mar 2023
Jul–Sep 2023
Jan–Mar 2024
Jul–Sep 2024

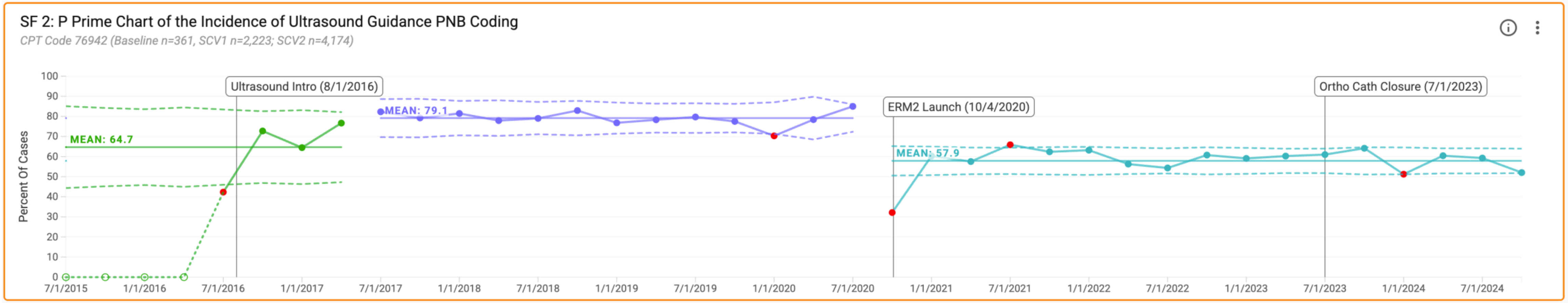
SF 2: P Prime Chart of the Incidence of Ultrasound Guidance PNB Coding
CPT Code 76942 (Baseline n=361, SCV1 n=2,223; SCV2 n=4,174)
Percent Of Cases
MEAN: 64.7
MEAN: 79.1
MEAN: 57.9
Ultrasound Intro (8/1/2016)
ERM2 Launch (10/4/2020)
Ortho Cath Closure (7/1/2023)
7/1/2015
1/1/2016
7/1/2016
1/1/2017
7/1/2017
1/1/2018
7/1/2018
1/1/2019
7/1/2019
1/1/2020
7/1/2020
1/1/2021
7/1/2021
1/1/2022
7/1/2022
1/1/2023
7/1/2023
1/1/2024
7/1/2024

SF 3A: X-bar Chart of the Average Anesthesia Prep Time with Regional Block

Anesthesia start to anesthesia ready time (Baseline n=1,341; SCV1 n=7,210; SCV2 n=3,732; SCV3 n=996)

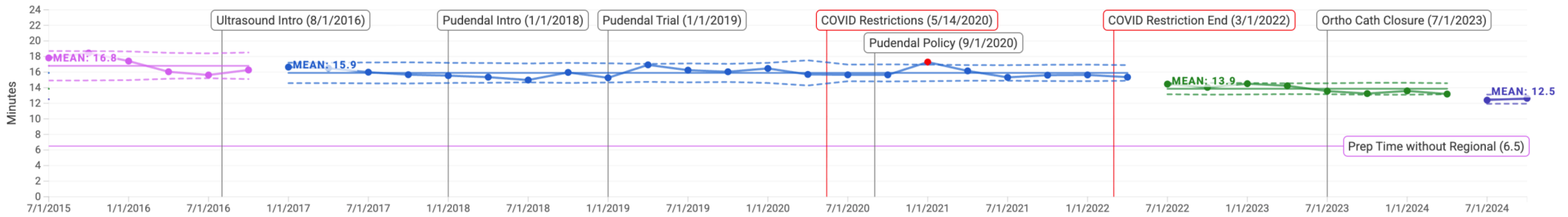

SF 3B: S-Chart of the Standard Deviation of Anesthesia Prep Time

Anesthesia start to anesthesia ready time (Baseline n=836; SCV1 n=4,380; SCV2 n=3,342; SCV3 n=2,398; SCV4 n=2,330)

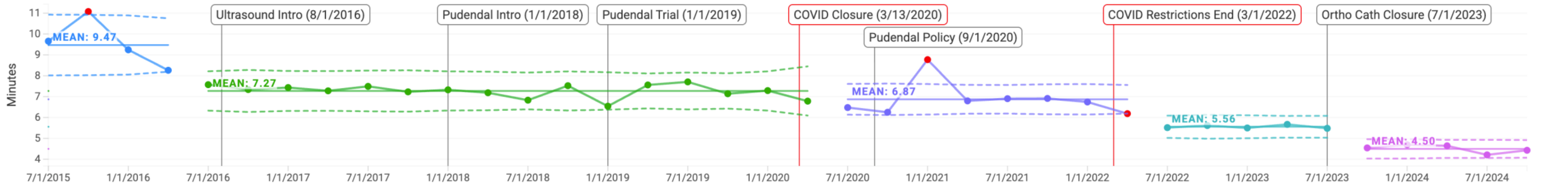

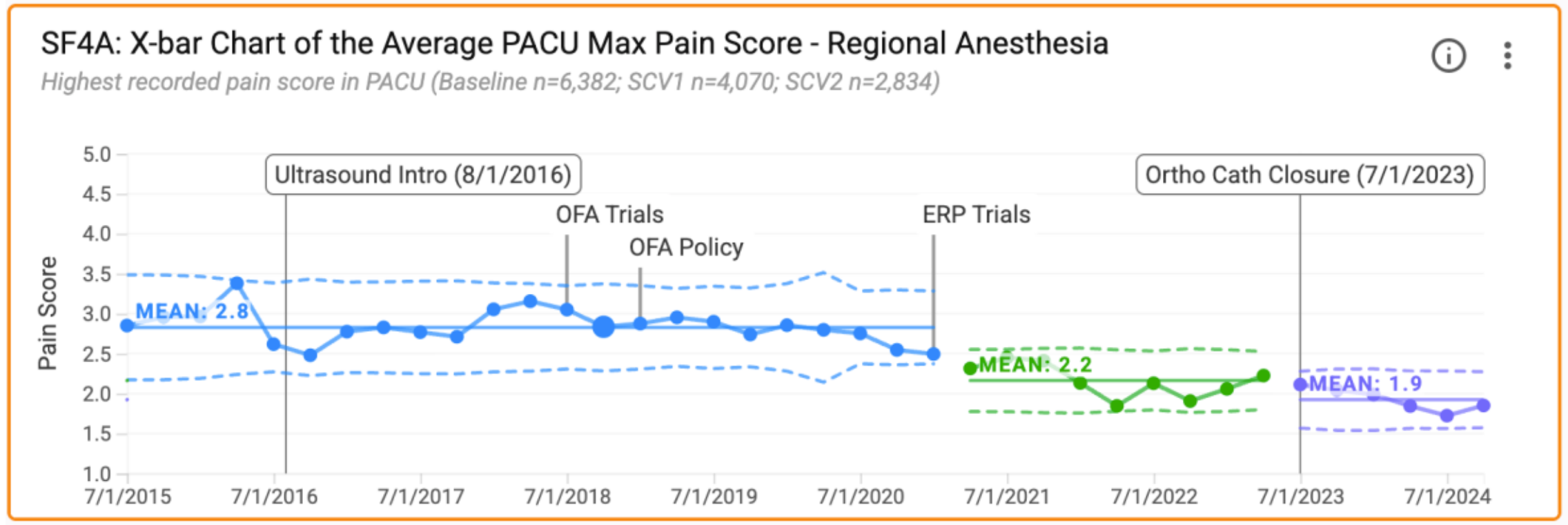
SF4A: X-bar Chart of the Average PACU Max Pain Score - Regional Anesthesia
Highest recorded pain score in PACU (Baseline n=6,382; SCV1 n=4,070; SCV2 n=2,834)
Pain Score
Ultrasound Intro (8/1/2016)
Ortho Cath Closure (7/1/2023)
OFA Trials
OFA Policy
ERP Trials
MEAN: 2.8
MEAN: 2.2
MEAN: 1.9
5.0 4.5 4.0 3.5 3.0 2.5 2.0 1.5 1.0
7/1/2015 7/1/2016 7/1/2017 7/1/2018 7/1/2019 7/1/2020 7/1/2021 7/1/2022 7/1/2023 7/1/2024

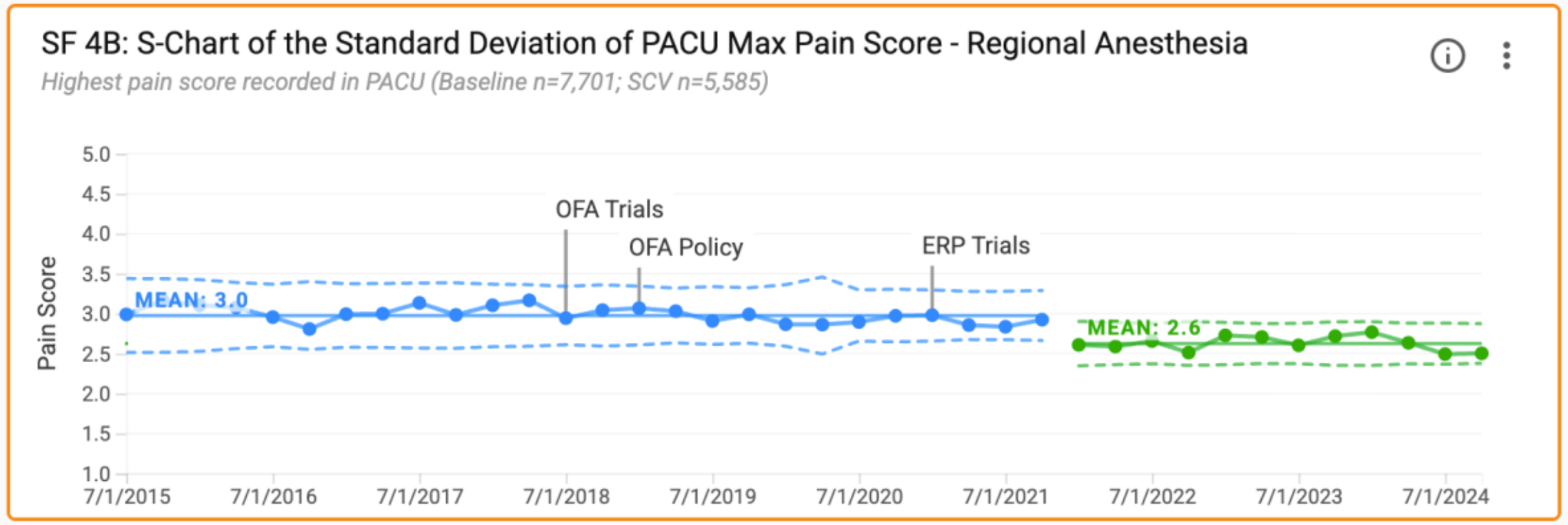
SF 4B: S-Chart of the Standard Deviation of PACU Max Pain Score - Regional Anesthesia
Highest pain score recorded in PACU (Baseline n=7,701; SCV n=5,585)
Pain Score
OFA Trials
OFA Policy
ERP Trials
MEAN: 3.0
MEAN: 2.6
5.0 4.5 4.0 3.5 3.0 2.5 2.0 1.5 1.0
7/1/2015 7/1/2016 7/1/2017 7/1/2018 7/1/2019 7/1/2020 7/1/2021 7/1/2022 7/1/2023 7/1/2024

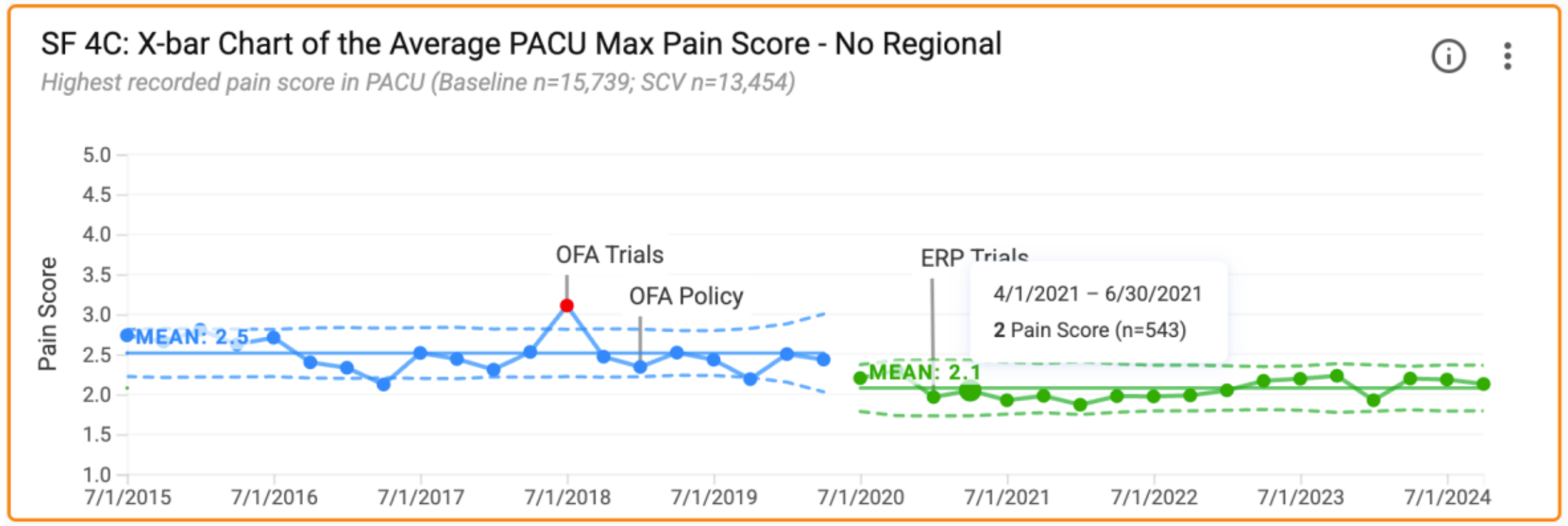
SF 4C: X-bar Chart of the Average PACU Max Pain Score - No Regional
Highest recorded pain score in PACU (Baseline n=15,739; SCV n=13,454)
Pain Score
OFA Trials
OFA Policy
ERP Trials
4/1/2021 – 6/30/2021
2 Pain Score (n=543)
MEAN: 2.5
MEAN: 2.1
5.0 4.5 4.0 3.5 3.0 2.5 2.0 1.5 1.0
7/1/2015 7/1/2016 7/1/2017 7/1/2018 7/1/2019 7/1/2020 7/1/2021 7/1/2022 7/1/2023 7/1/2024

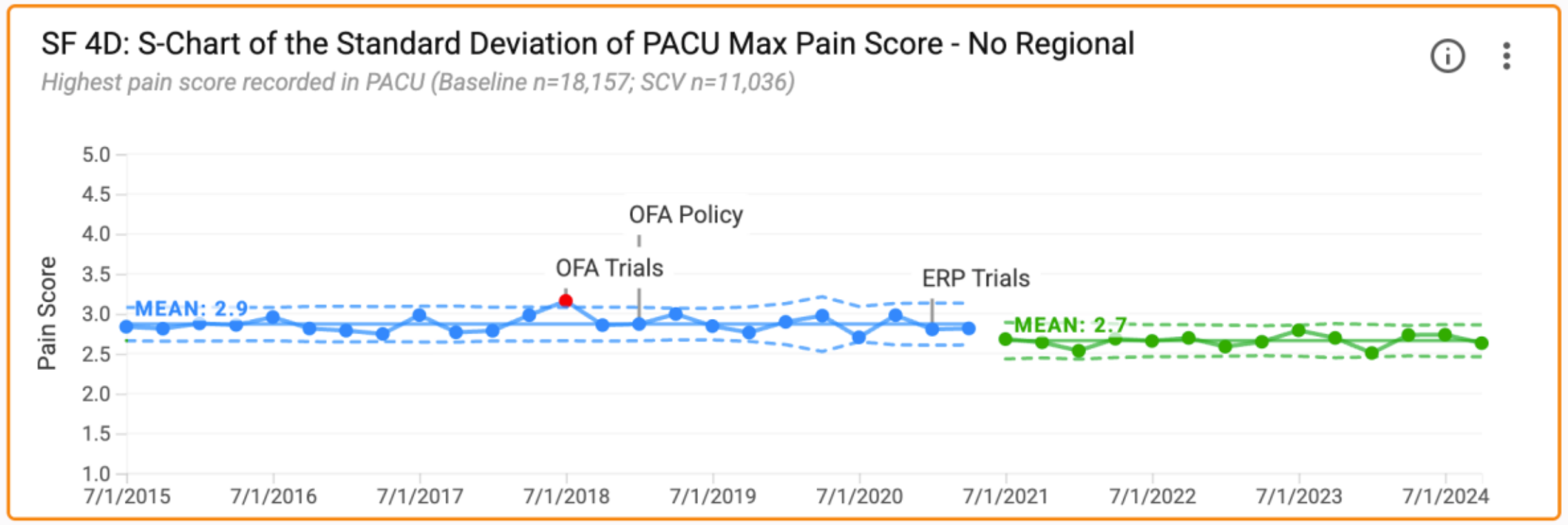
SF 4D: S-Chart of the Standard Deviation of PACU Max Pain Score - No Regional
Highest pain score recorded in PACU (Baseline n=18,157; SCV n=11,036)
Pain Score
OFA Policy
OFA Trials
ERP Trials
MEAN: 2.9
MEAN: 2.7
5.0 4.5 4.0 3.5 3.0 2.5 2.0 1.5 1.0
7/1/2015 7/1/2016 7/1/2017 7/1/2018 7/1/2019 7/1/2020 7/1/2021 7/1/2022 7/1/2023 7/1/2024

SF 5A: P-Chart of the IV Opioids Given in PACU Rate - Regional Anesthesia

Administration IV Opioids in PACU (Baseline n=3,495; SCV1 n=3,335; SCV2 n=3,622; SCV3 n=2,834)

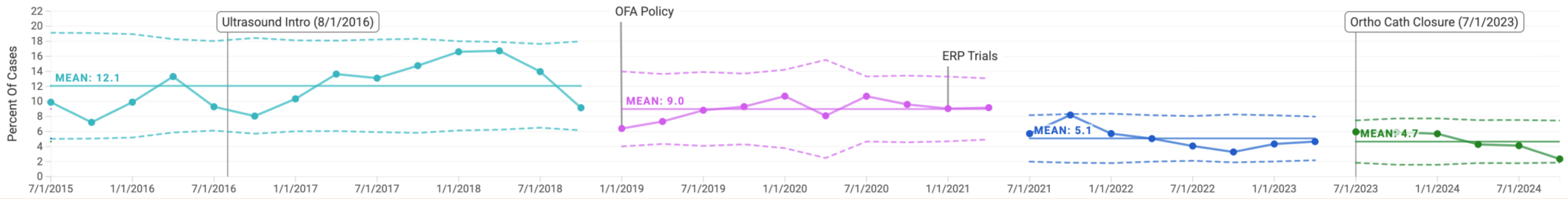

SF5B: P-Chart of the IV Opioids Given in PACU Rate - No Regional

Administration IV Opioids in PACU (Baseline n=11,320; NR SCV1 n=7,477; NR SCV2 n=10,396)

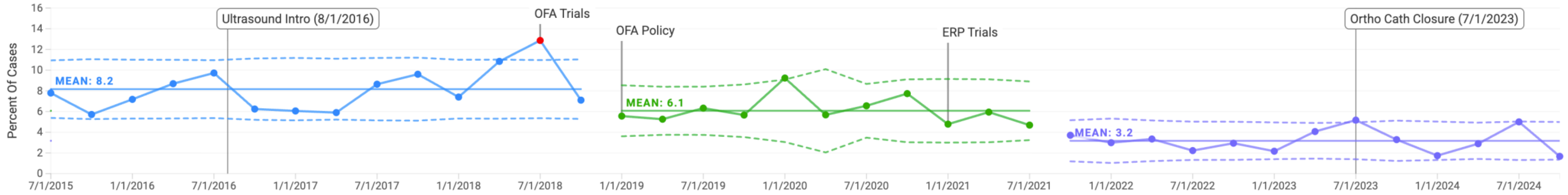

Supplement: Supplementary file 1 [file PAN-36-1341-s004.pdf]
