# Supplementary material for: Perspectives in Pediatric Ambulatory Anesthesia: Part 3—One Center's Evolving Regional Anesthesia Experience and Outcomes
Source: Paediatr Anaesth. 2026 Jul 9;36(11):1341–51. doi: 10.1002/pan.70255 (PMC13639202; doi:10.1002/pan.70255)
Supplement: Supplementary file 2 [file PAN-36-1341-s001.docx]

| **Supplemental Table 3: Ropivacaine Maximum Dose** | |
| --- | --- |
| **Ropivacaine 0.2% Maximum Dose** | |
| Patient Weight (Kg) | Ropivacaine 0.2% Max Volume (ml) |
| 4 | 6 |
| 6 | 9 |
| 8 | 12 |
| 10 | 15 |
| 12 | 18 |
| 14 | 21 |
| 16 | 24 |
| 18 | 27 |
| 20 | 30 |
| 22 | 33 |
| 24 | 36 |
| 26 | 39 |
| 28 | 42 |
| 30 | 45 |
| 32 | 48 |
| 34 | 51 |
| 36 | 54 |
| 38 | 57 |
| 40 | 60 |
| 42 | 63 |
| 44 | 66 |
| 46 | 69 |
| 48 | 72 |
| 50 | 75 |
| 52 | 78 |
| 54 | 81 |
| 56 | 84 |
| 58 | 87 |
| 60 | 90 |
| 62 | 93 |
| 64 | 96 |
| 66 | 99 |
| 68 | 102 |
| 70 | 105 |
|  | |
| **Ropivacaine 0.5% Maximum Dose** | |
| Patient Weight (Kg) | Ropivacaine 0.5% Max Volume (ml) |
| 4 | 2 |
| 6 | 3 |
| 8 | 4 |
| 10 | 6 |
| 12 | 7 |
| 14 | 8 |
| 16 | 9 |
| 18 | 10 |
| 20 | 12 |
| 22 | 13 |
| 24 | 14 |
| 26 | 15 |
| 28 | 16 |
| 30 | 18 |
| 32 | 19 |
| 34 | 20 |
| 36 | 21 |
| 38 | 22 |
| 40 | 24 |
| 42 | 25 |
| 44 | 26 |
| 46 | 27 |
| 48 | 28 |
| 50 | 30 |
| 52 | 31 |
| 54 | 32 |
| 56 | 33 |
| 58 | 34 |
| 60 | 36 |
| 62 | 37 |
| 64 | 38 |
| 66 | 39 |
| 68 | 40 |
| 70 | 42 |
