# Supplementary material for: Perspectives in Pediatric Ambulatory Anesthesia: Part 3—One Center's Evolving Regional Anesthesia Experience and Outcomes
Source: Paediatr Anaesth. 2026 Jul 9;36(11):1341–51. doi: 10.1002/pan.70255 (PMC13639202; doi:10.1002/pan.70255)
Supplement: Supplementary file 3 [file PAN-36-1341-s002.docx]

| **Supplemental Table 1: Regional anesthesia timeout checklist (version 2)** | |
| --- | --- |
| **CHALLENGE (RN only)** | **RESPONSE** |
| Patient ID | RN reads wrist band  Attending reads surgery consent  CRNA reads computer banner |
| Consent | Attending reads surgical consent |
| Site | All present confirm site markings |
| Block Type | Attending states block(s) |
| Allergies | Yes (state) / None |
| Max Dose | Verifies concentration to be used  States max dose from job aid |
| Infusion | Q-ball ordered / NA |
