# Supplementary material for: Perspectives in Pediatric Ambulatory Anesthesia: Part 3—One Center's Evolving Regional Anesthesia Experience and Outcomes
Source: Paediatr Anaesth. 2026 Jul 9;36(11):1341–51. doi: 10.1002/pan.70255 (PMC13639202; doi:10.1002/pan.70255)
Supplement: Supplementary file 4 [file PAN-36-1341-s003.docx]

| **Supplemental Table 2: Regional anesthesia timeout checklist (current version)** | | | |
| --- | --- | --- | --- |
| **Initiate checklist once all block set-up actives are complete** | | | |
| **CHALLENGE**  **(RN)** | **RESPONSE** | | |
|  | Anesthesiologist | CRNA / Fellow | Anesthesia Tech |
| Patient ID | matches consent | matches computer | Matches ultrasound |
| Surgical Site | Surgical site  matches consent |  |  |
| Block Type | States block(s) |  |  |
| Block Site | IDs and marks  block site(s) | Confirms | Confirms |
| Allergies |  | Y/N (specify) |  |
| Anticoagulation |  | Y/N (specify) |  |
| Skin Prep Restriction |  | Y/N (specify) |  |
| Medication | State ropivacaine (%)  and dose volume | RN: Confirm drug vial % & expire date;  Max dose verified |  |
|  | Draw up medication | |  |
| Infusion |  | Q-ball ordered / NA |  |
| Concerns? | Anyone state any concern | | |
